# Rise of *Neisseria gonorrhoeae* with mosaic *penA-34,* France, 2018–2023

**DOI:** 10.1128/aac.01919-25

**Published:** 2026-05-06

**Authors:** François Camelena, Manel Merimèche, Mary Mainardis, Aymeric Braille, Béatrice Bercot, ENGON Study Group, Agathe Goubard

**Affiliations:** 1 Laboratoire de Microbiologie, Institut Alfred Fournier, Paris, France; 2 Laboratoire de Bactériologie, Centre Hospitalier Jura Sud, Lons-le-Saunier, France; 3 Laboratoire de Microbiologie, CHRU Nancy, Nancy, France; 4 Laboratoire de Microbiologie, Hôpital Paris Saint Joseph, Paris, France; 5 Laboratoire de Microbiologie, LABM Labouest, Trelaze, France; 6 Laboratoire de Microbiologie, CH Saint-Nazaire, Saint-Nazaire, France; 7 Laboratoire de Microbiologie, CH Rochefort, Rochefort, France; 8 Laboratoire de Bactériologie, CHU Besançon, Besançon, France; 9 Laboratoire de Microbiologie, Hôpital Robert Debré, APHP, Paris, France; 10 Laboratoire Départemental de Biologie Médicale de Bondy, Bondy, France; 11 Laboratoire de Microbiologie, LABM ABO+, Chambray-les-tours, France; 12 Laboratoire de Microbiologie, Hôpital Ambroise Paré, APHP, Paris, France; 13 Laboratoire de Bactériologie, CHU de Poitiers, Poitiers, France; 14 Laboratoire de Microbiologie, CHI Alpes du Sud, Gap, France; 15 Laboratoire de Microbiologie, LABM MS BIO POLE ANTILLES, Baie-Mahaut, France; 16 Laboratoire de Bactériologie, Hôpital Cochin, APHP, Paris, France; 17 Laboratoire de Microbiologie, CH Guéret, Guéret, France; 18 Laboratoire de Microbiologie, LABM BIO67, Strasbourg, France; 19 Laboratoire de Microbiologie, LABM CERBALLIANCE NORMANDIE, Lisieux, France; 20 Laboratoire de Microbiologie, CH Laval, Laval, France; 21 Laboratoire de Microbiologie, LABM des Andaines - Normandie Maine, La Ferté-Macé, France; 22 Laboratoire de Microbiologie, LABM MEDIBIOLAB, Saran, France; 23 Laboratoire de Bactériologie, CHU de Lille, Lille, France; 24 Laboratoire de Bactériologie, CHU Bordeaux, Bordeaux, France; 25 Laboratoire de Bactériologie, CHU Strasbourg, Strasbourg, France; 26 Laboratoire de Microbiologie, LABM Novabio, Notre-Dame-de-Sanilhac, France; 27 Laboratoire de Microbiologie, CH Drome Nord, Romans sur Isère, France; 28 Laboratoire de Bactériologie, CHU de Caen, Caen, France; 29 Laboratoire de Bactériologie, CHU d'Orléans, Orléans, France; 30 Laboratoire de Bactériologie, CHU Nancy, Nancy, France; 31 Laboratoire de Bactériologie, CHU de Lyon, Lyon, France; 32 Laboratoire de Bactériologie, CHU de Toulouse, Toulouse, France; 33 Laboratoire de Microbiologie, EXALAB, Le Haillan, France; 34 Laboratoire de Microbiologie, CH Métropole Savoie, Chambéry, France; 35 Laboratoire de Bactériologie, Hôpital Bichat, APHP, Paris, France; 36 Laboratoire de Bactériologie, Hôpital Necker, APHP, Paris, France; 37 Laboratoire de Microbiologie, CH Foch, Suresnes, France; 38 Laboratoire de Microbiologie, LABM DYNALAB, Romilly-sur-Seine, France; 39 Laboratoire de Microbiologie, LABM UNILABS, Chalon Sur Saône, France; 40 Laboratoire de Microbiologie, CHU Limoges, Limoges, France; 41 Laboratoire de Microbiologie, LABM BIOCEANE, Le Havre, France; 42 Laboratoire de Microbiologie, CH MOULINS-YZEURE, Moulins-Yzeure, France; 43 Laboratoire de Microbiologie, LABM Astralab, Limoges, France; 44 Laboratoire de Bactériologie, Hôpitaux Saint-Louis - Lariboisière, APHP, Paris, France; 45 Laboratoire de Bactériologie, CHU de Strasbourg, Strasbourg, France; 46 Laboratoire de Bactériologie, CHU Rennes, Rennes, France; 47 Laboratoire de Bactériologie, Hôpital Saint-Antoine, APHP, Paris, France; 48 Laboratoire de Bactériologie, CHU Brest, Brest, France; 49 Laboratoire de Microbiologie, LABM Laborizon BIORYLIS, La Roche Sur Yon, France; 50 Laboratoire de Microbiologie, LABM Bioexcel, Saint Doulchard, France; 51 Laboratoire de Microbiologie, CH Public du Cotentin, Cherbourg, France; 52 Laboratoire de Microbiologie, CH Toulon, Toulon, France; 53 Laboratoire de Bactériologie, Hôpital Louis Mourier, APHP, Paris, France; 54 Laboratoire de Bactériologie, Hôpital Henri Mondor, Créteils, France; 55 Laboratoire de Microbiologie, Hôpital Beaujon, APHP, Clichy, France; 56 Laboratoire de Bactériologie, CHU Angers, Angers, France; 57 Laboratoire de Bactériologie, CHU Grenoble, Grenoble, France; 58 Laboratoire de Microbiologie, CH Aix-en-Provence, Aix-en-Provence, France; 59 Laboratoire de Microbiologie, LABM Inovie Labosud, Montpellier, France; 60 Laboratoire de Microbiologie, BIOLAM LCD, Saint-Denis, France; 61 Laboratoire de Bactériologie, CHU Reims, Reims, France; 62 Laboratoire de Bactériologie, CHU Clermont-Ferrand, Clermont-Ferrand, France; 63 Laboratoire de Microbiologie, LABM LDA13, Marseille, France; 64 Laboratoire de Microbiologie, CH Argenteuil, Argenteuil, France; 65 Laboratoire de Bactériologie, Hôpital Pitié Salpétrière, APHP, Paris, France; 66 Laboratoire de Microbiologie, Hôpital Kremlin Bicêtre, APHP, Kremlin Bicêtre, France; 67 Laboratoire de Microbiologie, Hôpital Louis Mourier, APHP, Paris, France; 68 Laboratoire de Microbiologie, CH Versailles, Versailles, France; 69 Laboratoire de Microbiologie, CHU de Saint-Etienne, Saint-Etienne, France; 70 Laboratoire de Microbiologie, CH Tahiti, Tahiti, France; 71 Laboratoire de Microbiologie, CH Flers, Flers, France; 72 Laboratoire de Microbiologie, CH Melun, Melun, France; 73 Laboratoire de Microbiologie, LABM CBM 25, Besançon, France; 74 Laboratoire de Microbiologie, LABM BIOPOLE 66, Cabestany, France; 75 Laboratoire de Bactériologie, CHU Tours, tours, France; 76 Laboratoire de Bactériologie, CHU Rouen, Rouen, France; 77 Laboratoire de Microbiologie, LABM Biomer, Metz, France; 78 Laboratoire de Bactériologie, Hôpital Antoine Beclère, APHP, Clamart, France; 79 Laboratoire de Microbiologie, LABM MEDILAB, Chauray, France; 80 Laboratoire de Bactériologie, CHU La Réunion, La Réunion, France; 81 Laboratoire de Microbiologie, CH Compiègne Noyon, Compiègne, France; 82 Laboratoire de Microbiologie, LABM Oriade-Noviale, Saint Martin d'Hères, France; 83 Laboratoire de Microbiologie, CH Niort, Niort, France; 84 Laboratoire de Microbiologie, CH Tourcoing, Tourcoing, France; 85 Laboratoire de Bactériologie, CHU Lille, Lille, France; 86 Laboratoire de Microbiologie, LABM BIOPAJ VAUBAN, Valenciennes, France; 87 Laboratoire de Microbiologie, CH Ouest Vosgien, Neufchateau, France; 88 Laboratoire de Bactériologie, CHU Nantes, Nantes, France; 89 Laboratoire de Microbiologie, LABM SELCO BIO, Selles-sur-Cher, France; 90 Laboratoire de Microbiologie, CH Boulogne sur Mer, Boulogne sur Mer, France; 91 Laboratoire de Microbiologie, CH Lens, Lens, France; 92 Laboratoire de Bactériologie, CHU Montpellier, Montpellier, France; 93 Laboratoire de Microbiologie, LABM Biolyss, Limoges, France; 94 Laboratoire de Microbiologie, CH de Lens, Lens, France; 95 Service de maladie Infectieuse, Hôpital Saint-Antoine, APHP, Paris, France; 96 Laboratoire de Bactériologie, CHU de Saint-Etienne, Saint-Etienne, France; 97 Laboratoire de Microbiologie, LABM SYNLAB SYLAB, Aurillac, France; 98 Laboratoire de Microbiologie, CH Saint Quentin, Saint Quentin, France; 99 Laboratoire de Microbiologie, LABM BIO 86, Poitiers, France; 100 Laboratoire de Microbiologie, LABM Bio Paris Ouest, Levallois Perret, France; 1 Laboratory of Bacteriology, Hospital Saint-Louis and Lariboisière, Assistance-Publique Hôpitaux de Paris 55663, Paris, France; 2 Associated Laboratory of the French National Centre for Bacterial Sexually Transmitted Infections, Saint Louis Hospital 728545, Paris, France; 3 Paris Cité University, Inserm, IAME 555089, Paris, France; The Peter Doherty Institute for Infection and Immunity, Melbourne, Victoria, Australia

**Keywords:** *gonorrhoeae*, mosaic *penA*, *penA-34*, genomic surveillance

## Abstract

Surveillance of antimicrobial resistance in *Neisseria gonorrhoeae* was conducted in France from 2018 to 2023. The proportion of isolates with cefixime MIC ≥ 0.032 mg/L increased from 8.1% in 2018–2022 to 23.5% in 2023. This sharp rise was driven by the expansion of two clones, ST1580 and ST16676, carrying the mosaic *penA-34.001* and *penA-34.007* alleles, respectively. These findings highlight the essential role of genomic surveillance in monitoring the evolution of resistance determinants in *N. gonorrhoeae*.

## INTRODUCTION

In *Neisseria gonorrhoeae* (NG), mosaic *penA* alleles, encoding penicillin-binding protein 2 (PBP2), are the main mechanism for decreased susceptibility and resistance to extended-spectrum cephalosporins (ESCs) (1). Since the early 2000s, resistance to cefixime has been associated with the mosaic *penA-10.001* allele, which was subsequently replaced by the mosaic *penA-34.001* allele (2, 3). This latter allele became the main cause of resistance to cefixime and decreased susceptibility to ceftriaxone in men who have sex with men (MSM), leading to the replacement of cefixime by ceftriaxone plus azithromycin for empirical gonorrhea therapy in Europe in 2012 (2). Since then, cefixime resistance has declined, accounting for only 0.5% of isolates in Europe in 2020. Cefixime remains recommended as an alternative regimen to treat uncomplicated NG infections in adults, particularly when intramuscular administration is contraindicated or refused, according to several guidelines (4–6). However, rising cefixime MICs may reduce its efficacy against NG (7, 8). In response to the increasing proportion of isolates exhibiting decreased susceptibility to ESCs, UK guidelines have recently revised the recommended cefixime regimen, increasing the dose from 400 mg single dose to 800 mg (400 mg twice daily) (4). Meanwhile, alarming reports from Southeast Asia and China describing a high prevalence of ESC resistance driven by mosaic *penA* alleles—mostly the *penA-60* allele—are worrying and further reinforce the need for strengthened epidemiological surveillance (9–11). In this study, we investigated the recent evolution of decreased susceptibility to cefixime in NG in France.

In France, NG *penA* allele diversity is monitored through annual whole-genome sequencing of isolates collected during a four-month nationwide prospective sentinel surveillance program (ENGON) involving voluntary public and private laboratories. The program is conducted by the National Reference Center for Bacterial Sexually Transmitted Infections for NG, Paris, France (NRC-NG), and received 2,966 isolates from 95 laboratories between 2018 and 2023, almost exclusively from mainland France (99.5% of isolates). Of these, about 80% were from men, and the median patient age was 28 years. Most isolates were obtained from urethral samples (49.8%), followed by vaginal swabs (17.3%), urine samples (17.1%), and anal swabs (10.8%).

We determined MICs of ceftriaxone, cefixime, azithromycin, spectinomycin, ciprofloxacin, and tetracycline using the Etest (bioMérieux) and interpreted using EUCAST guidelines for all isolates sent to the NRC-NG.

**Fig 1:**
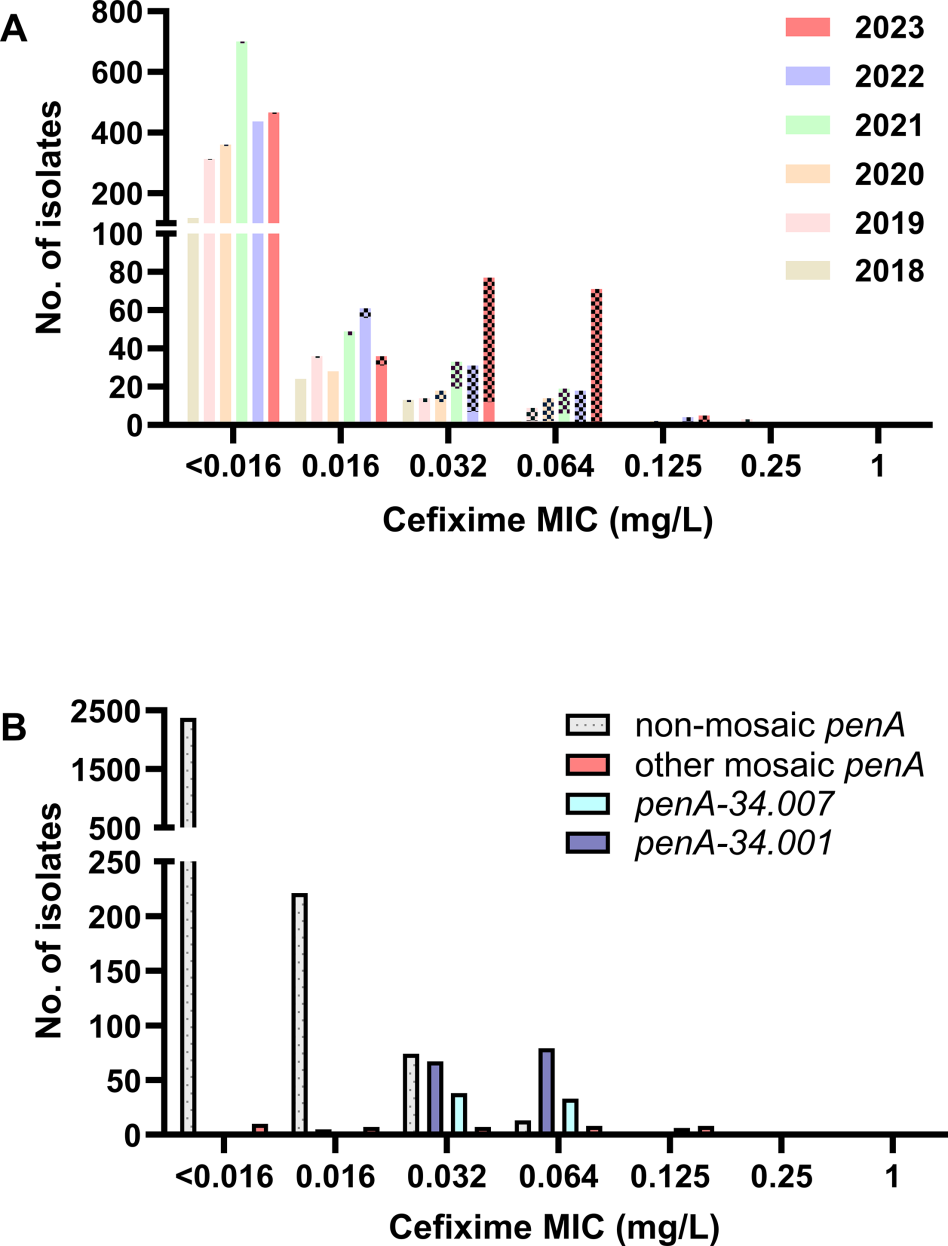
Distribution of MICs to cefixime among *Neisseria gonorrhoeae* isolates, France, 2018–2023. (**A**) Distribution of MICs to cefixime by year. Cross-hatched bars indicate isolates harboring mosaic *penA* alleles. (**B**) Distribution of MICs to cefixime by *penA* allele type, France, 2018–2023.

The proportion of isolates with cefixime MICs ≥ 0.032 mg/L increased significantly from 8.1% (186/2,310) in 2018–2022 to 23.5% (154/656) in 2023 (*P* < 0.0001, χ² test). The distribution of cefixime MICs is shown in Fig. 1.

**Fig 2:**
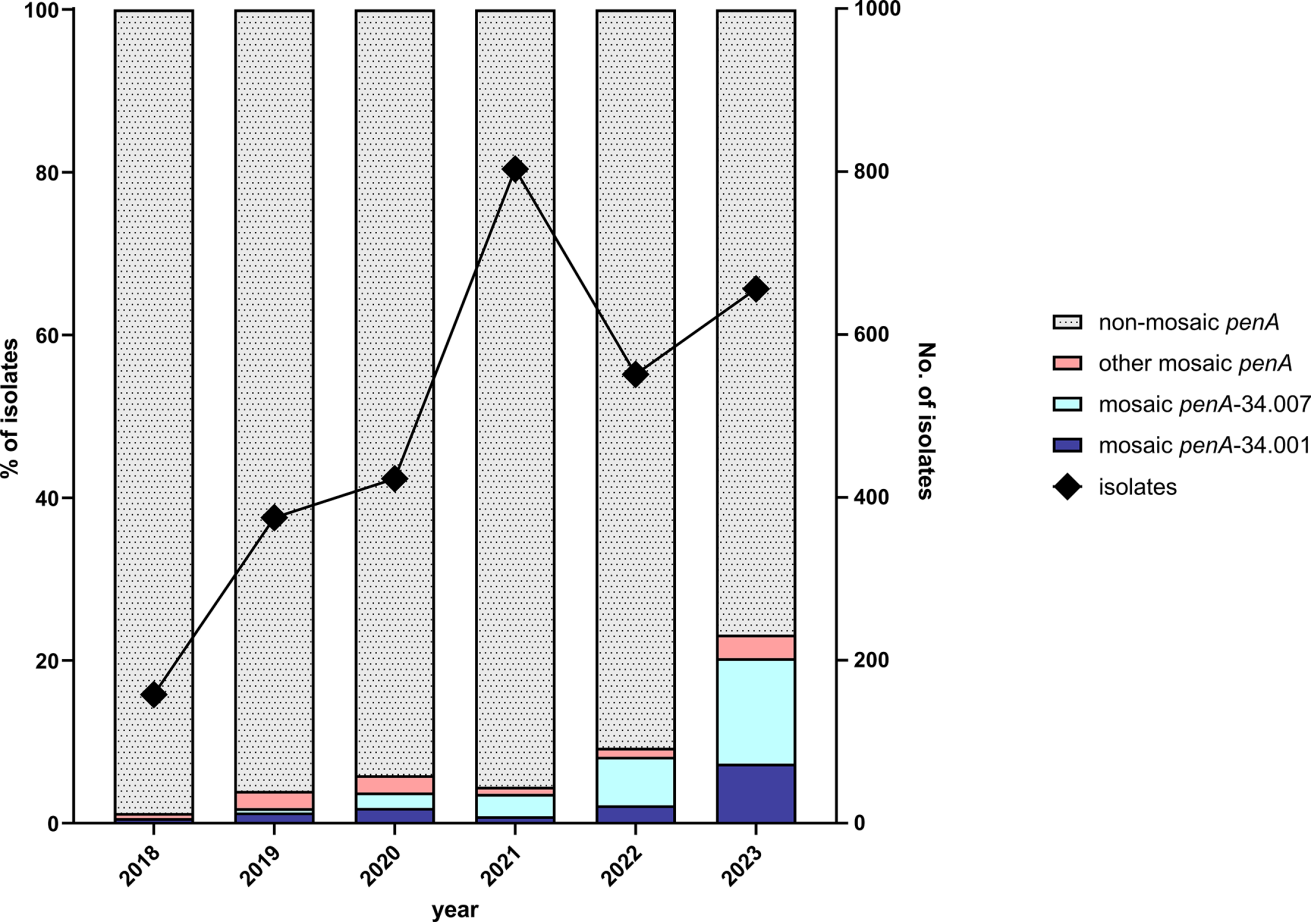
Distribution of *penA* alleles in isolates by year, France, 2018–2023. Bar histograms represent the annual distribution (in percentage) of *penA* allele types, while diamonds indicate the number of isolates analysed each year.

All isolates collected from the national surveillance program were sequenced using the Illumina platform and analysed as previously described (12). Among the 2,966 NG analysed, 45 distinct *penA* alleles were identified, including 15 mosaic alleles. A cefixime MIC  ≥ 0.032  mg/L was observed in 89.7% (252/281) of isolates carrying a mosaic *penA* allele. Mosaic *penA* isolates represented 74.1% (252/340) and 90.9% (140/154) of all isolates with a cefixime MIC ≥ 0.032 mg/L and ≥ 0.064 mg/L, respectively (Fig. 1). From 2018 to 2023, the prevalence of mosaic *penA* alleles increased significantly from 1.3% (2/158) in 2018 to 23.2% (152/656) in 2023, largely driven by a marked increase in the *penA-34* alleles in 2023 (*χ*² test, *P* < 0.0001) (Fig. 2). The most prevalent mosaic allele was *pen*A-*34.007* (54.8%, 154/281), followed by *penA-34.001* (28.8%, 81/281), *penA-93.001* (3.6%, 10/281)), and *penA-10.001* (2.8%, 8/281).

Interestingly, *penA-34.007* was first detected in 2019 and became the most frequent mosaic allele in 2021. This allele was initially identified in three multilocus sequence type (MLST) clones, ST7363, ST9362, and ST8123, which represented 87% of all *penA-34.007* isolates until 2023, when a new ST16676 clone emerged. The ST16676 clone harbored the *penA-34.007* allele in 90.2% (55/61) of isolates, while the remaining isolates exhibited a non-mosaic *penA-14.001* allele. This clone was first identified in France in 2023, and a few isolates have also been reported in Canada, the United Kingdom, and Norway since 2022 (13). It accounted for 9.3% (61/656) of all the NG isolates and 64.7% (55/85) of the *penA-34.007* identified from the national surveillance program in 2023. This clone displayed an azithromycin MIC ≤ 1 mg/L and was resistant to ciprofloxacin due to mutations in GyrA (S91F and D95A) and ParC (S87R). It also harbored the *bla*_TEM-1_ and *tetM* genes, which confer resistance to penicillin and high-level resistance to tetracycline (including doxycycline), respectively. ST16676 isolates were found only in males and were distributed throughout France with most cases identified in Paris area (59.0%, 36/61).

The second most common allele, *penA-34.001*, was identified in our collection in 2018 and remained the most prevalent until 2020. This allele was identified in four MLST clones: ST1580, ST1901, ST1902, and ST9362. However, the ST1580 isolates were largely dominant, representing 77.1% (64/83) of all isolates harboring *penA-34.001* and 98.0% (47/48) of those isolated in 2023. This clone harbored mosaic *penA* in 98.7% (74/75) of isolates, including mostly *penA-34.001* (64/75, 85.3%). The ST1580 isolates with *penA-34.001* were resistant to ciprofloxacin, tetracycline, and azithromycin in 92.1% (59/64), 100% (64/64), and 21.9% (14/64), respectively. This clone was predominantly detected in males (95.3%, 61/64) across France, with most cases similarly originating from the Paris area (53.1%, 34/64).

**Fig 3:**
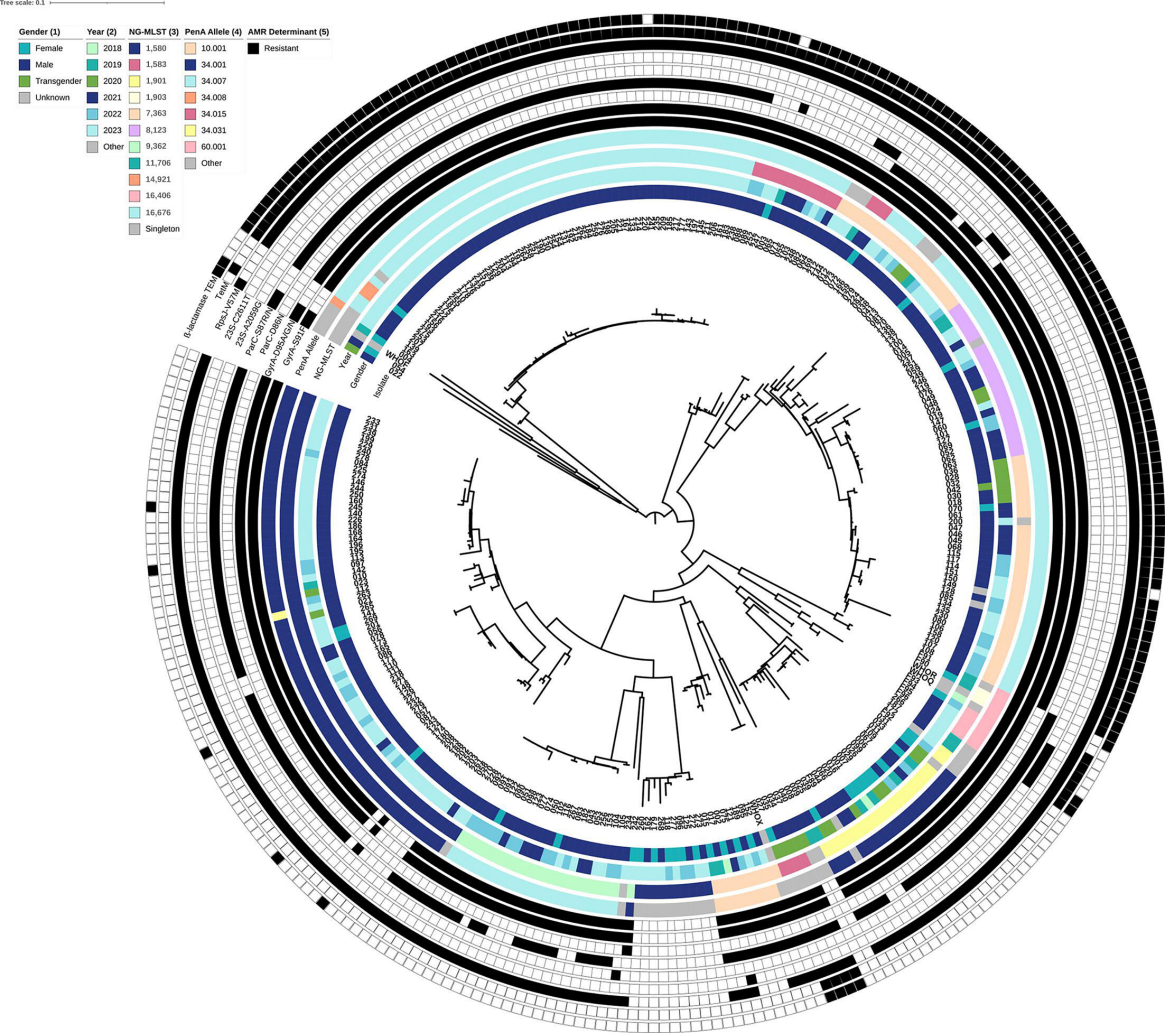
Phylogenomic tree of *Neisseria gonorrhoeae* isolates harboring mosaic *penA* alleles, France, 2018–2023. The tree was constructed using 26,238 core single-nucleotide polymorphism positions. Mosaic *penA* isolates from the ENGON annual surveillance programs were compared with reference strains (WHOF, WHOQ, WHOR, WHOX, and WHOY), ceftriaxone-resistant strains (F90, F91, and F92), and extensively drug-resistant strains (F93, F94, F95, and F96). The scale bar indicates branch length corresponding to genetic distance.

Analysis of the single nucleotide polymorphism (SNP)-based core genome phylogeny of mosaic *penA* isolates revealed a structured population consisting of multiple lineages and sublineages. Notably, ST16676 belonged to a distinct lineage, separate from other major clones, including ST1580, ST7363, ST9362, and ST8123. The ST16676 isolates exhibited a median pairwise SNP distance of 116 (range: 0–967), indicating a highly clonal population. In contrast, ST1580 showed a median pairwise SNP distance of 1,202 (range: 0–4,278), reflecting greater genetic diversity, consistent with the multiple resistance genotypes observed (Fig. 3).

These distribution patterns may reflect transmission dynamics within specific sexual networks, particularly among MSM. Notably, NG strains circulating among MSM have been reported to exhibit higher resistance than those found in the heterosexual population (14, 15). Recent data from the DOXYVAC trial showed that infections caused by NG harboring *penA-34.007* and *tet(M*) were more frequent among MSM receiving doxycycline postexposure prophylaxis (PEP) than among those not receiving PEP (16).

Although isolates harboring the *penA-34* alleles remain susceptible to ceftriaxone, this allele is similar to *penA-42* and differs by only one SNP leading to the A501P substitution in PBP2, which has been associated with ceftriaxone resistance (17). By contrast, ceftriaxone resistance is now primarily attributable to the *penA-60* allele (95% sequence identity with *penA-34*), which encodes a PBP2 variant notably carrying the A311V substitution—the most frequently reported molecular marker of ceftriaxone resistance—and this substitution is not present in PenA-34 (18).

A limitation of this study is that the exact national coverage of the NG population circulating in France by the ENGON surveillance program remains unknown. In addition, the representativeness of the isolates might be affected by a bias toward urethral samples, as NG is more easily recovered from these specimens in culture. Nevertheless, the network provides a broad overview of circulating strains and resistance trends.

The cause of the recent emergence and expansion of mosaic *penA* isolates remains unclear. The lack of systematic data, such as HIV status and the use of doxycycline post-exposure prophylaxis, prevented the identification of risk factors associated with these mosaic *penA* isolates. The spectacular rise of these clones highlights the ability of the gonococcal population to rapidly shift and reshape the epidemiology of antimicrobial resistance. Considering the reported epidemiological situation in Southeast Asia and China, the sudden spread of clones harboring *penA-60* allele represents a plausible and concerning scenario. Overall, these observations emphasize the importance of combining MIC testing with molecular surveillance to enable the early detection and effective monitoring of such high-risk clones.

These results highlight the critical need for sustained public health efforts to monitor the spread of antimicrobial resistance in NG in order to adapt treatment guidelines in line with emerging resistance patterns.

## Data Availability

Whole genome sequencing (WGS) data from the 281 French mosaic *penA* isolates collected between 2018 and 2023 are available under NCBI BioProject numbers PRJNA1302729 and PRJNA1082518 (F95 isolate) and PRJNA998219 (F93 isolate).

## AFTER EPUB

*[This article was published on 6 May 2026 with an error in the byline. The byline, and accordingly the contributors, were corrected in the current version, posted on 20 July 2026.]*
